# In a Bilingual Mood: Mood Affects Lexico-Semantic Processing Differently in Native and Non-Native Languages

**DOI:** 10.3390/brainsci12030316

**Published:** 2022-02-26

**Authors:** Marcin Naranowicz, Katarzyna Jankowiak, Patrycja Kakuba, Katarzyna Bromberek-Dyzman, Guillaume Thierry

**Affiliations:** 1 Faculty of English, Adam Mickiewicz University, 61712 Poznań, Poland; katarzyna.jankowiak@amu.edu.pl (K.J.); patrycja.kakuba@amu.edu.pl (P.K.); kabrod@amu.edu.pl (K.B.-D.); g.thierry@bangor.ac.uk (G.T.); 2 School of Human and Behavioural Sciences, Bangor University, Bangor LL57 2DG, UK

**Keywords:** bilingualism, mood, lexico-semantic processing, emotion regulation, meaning integration, event-related potentials

## Abstract

Positive and negative moods tend to have differential effects on lexico-semantic processing in the native language (L1). Though accumulating evidence points to dampened sensitivity to affective stimuli in the non-native language (L2), little is known about the effects of positive and negative moods on L2 processing. Here, we show that lexico-semantic processing is differently affected by positive and negative moods only in L1. Unbalanced Polish–English bilinguals made meaningfulness judgments on L1 and L2 sentences during two EEG recording sessions featuring either positive- or negative-mood-inducing films. We observed a reduced N1 (lexical processing) for negative compared to positive mood in L2 only, a reduced N2 (lexico-semantic processing) in negative compared to positive mood in L1 only, a reduced N400 (lexico-semantic processing) for meaningless compared to meaningful L1 sentences in positive mood only, and an enhanced late positive complex (semantic integration and re-analysis) for L2 compared to L1 meaningful sentence in negative mood only. Altogether, these results suggest that positive and negative moods affect lexical, lexico-semantic, and semantic processing differently in L1 and L2. Our observations are consistent with previous accounts of mood-dependent processing and emotion down-regulation observed in bilinguals.

## 1. Introduction

Affect (i.e., emotions, attitudes, feelings, and moods) permeates all aspects of human existence, including communicative interactions, oftentimes unobtrusively yet pervasively [1]. Seeing that 21st century communication is incrementally becoming international, with most people around the world speaking more than one language daily [2], it seems vital to shift research attention towards the relationship between affect and bilingualism. Unbalanced bilinguals, for instance, have frequently been observed to show dampened emotional sensitivity to non-native content (see [3] for a review). Interestingly, recent evidence has also shown that emotional word processing can also be affected by mood, a current affective background state [4,5]. However, mood effects on language comprehension in a broader communicative context in bilinguals have received little scholarly attention. The present study thus investigates potential differences in positive and negative mood effects on lexico-semantic processing in native (L1) and non-native (L2) languages.

### 1.1. Emotion Effects on Bilingualism

There is a growing interest in the relationship between affect and language nativeness, with emotion research showing both similarities and differences between L1 and L2 (see [3] for a review). However, recent evidence has more often pointed to dampened affective sensitivity in L2, especially in relation to negatively valenced content, as indexed by survey [6,7], behavioural [8,9], physiological [10,11], electrophysiological [12,13], and hemodynamic responses [14,15]. For instance, in an event-related potential (ERP) study, Jończyk et al. [13] observed a reduced N400 response to negative compared to positive L2 sentences and compared to positive and negative L1 sentences in immersed Polish–English bilinguals. Overall, such emotional detachment in L2 in unbalanced late bilinguals has been associated with learning L2 in an instructional (i.e., not immersive) environment [6], a late age of L2 acquisition combined with low L2 proficiency [16], and a weaker connection between lexico-semantic representations and affect in L2 due to infrequent use of emotional words [17].

Recent evidence has also shown that when emotionally evocative stimuli are not language-bound, bilinguals are able to implicitly down-regulate the magnitude of their emotional response more effectively in L2 than L1 [18,19]. For instance, Morawetz et al. [18] observed a more effective implicit emotion regulation through content labelling (i.e., choosing a noun semantically related to a presented picture) in participants’ L2 (German) than in their L1 (English). Such results suggest that bilinguals can effectively activate automatic emotion regulatory strategies when using their L2. It remains an open question, however, whether L2 comprehension facilitates such regulation efforts to the same degree as L2 production.

### 1.2. Mood and Semantic Processes in L1

Mood has been defined as an unobtrusive, slowly changing, and low-intensity affective background state, fluctuating across time from feeling good (a positive mood) to feeling bad (a negative mood) [20]. Mood effects on language have been studied employing the N400 component: a negativity with a centro-parietal scalp distribution, peaking in amplitude at around 300–500 ms post stimulus onset [21]. It is often referred to as an anomaly detector; increased N400 amplitudes can, for instance, be evoked by critical words semantically incongruent with a sentence context [22]. Two other language-related ERP components have shown sensitivity to mood changes: N1 [4] and the late positive complex (LPC) [23]. N1 is a negativity peaking at around 100–200 ms post stimulus onset over parietal electrodes [24]. Besides being sensitive to word lexical characteristics (e.g., lexical frequency) [25], N1 amplitudes can also be altered when socially relevant feedback is anticipated [26] and by positive and negative moods [4]. LPC is a positive-going brainwave peaking at around 600–800 ms over centro-parietal electrodes [24]. An increase in LPC amplitude has been associated with more controlled, higher-level (cognitive) stimulus processing and the re-allocation of attentional, motivational, and evaluative resources [27].

Electrophysiological research on monolinguals has pointed to qualitatively different modulatory effects of positive and negative moods on semantic processes [1,23,28,29]. For instance, Chwilla et al. [23] presented participants with positive and negative film clips and asked them to read high-cloze (i.e., semantically correct) and low-cloze (i.e., semantically anomalous) sentences. They observed increased N400 amplitudes for low-cloze compared to high-cloze sentences in participants experiencing a positive mood, with the effect being restricted to the right hemisphere and left occipital and temporal sites in the negative mood condition. According to the authors, these results suggest that a positive and negative mood do not lead to quantitative differences in cognitive processing (e.g., a relative decrease in motivation or attention in a negative mood) but to qualitatively different lexico-semantic processing styles [28,29].

Furthermore, Pinheiro et al. [28] presented participants with positive, negative, and neutral pictures and asked them to make semantic judgements about sentence pairs ending in an (i) expected word, (ii) unexpected word of the same semantic category (i.e., within-category violation), and (iii) unexpected word from a different yet semantically related category (i.e., between-category violation). Whilst within-category violations resulted in a more pronounced N400 response to the negative mood condition, the positive mood condition evoked decreased N400 amplitudes, suggesting that negative and positive moods tend to weaken and strengthen, respectively, lexico-semantic access to words within a given semantic network. Crucially, the authors also found that expected items elicited attenuated N400 amplitudes in the negative compared to the positive mood condition, which points to an increased word expectancy in the negative mood condition, possibly due to a higher sensitivity to contextual information and more attention to detail.

### 1.3. Mood and Semantic Processes in L2

The modulation of language comprehension by mood has recently been studied in the bilingual context. Naranowicz et al. [5] asked unbalanced Polish–English bilinguals to watch positive and negative film clips and then classify single words as positive, negative, or neutral. Unlike males, females categorised the stimuli faster in a positive compared to negative mood. While there was no between-mood difference in response times to L1 positive words, participants responded faster to L2 positive words when they were in a positive compared to negative mood. Additionally, participants categorised neutral words faster in L1 than L2 in the positive mood condition only.

Similarly, Kissler and Bromberek-Dyzman [4] asked unbalanced German–English bilinguals to watch mood-inducing film clips and categorise decontextualised words as positive, negative, or neutral in an ERP study. They observed an attenuated left-lateralised N1 response to L1 words in the positive compared to the negative mood condition but no between-mood difference for L2 words, suggesting that the effects of mood on the early stage of word processing might be limited to L1. However, they found no mood–language interaction in either the N400 or the LCP time windows, which points to a possibly short-lived effect of mood induction in L1 and L2, at least in the case of single word processing. Altogether, bilingual research has shown that emotional word processing can be differently modulated by positive and negative moods, at least at the initial stages of individual word processing. To our knowledge, there is no tangible evidence to date regarding the effect of positive and negative moods on L1 and L2 semantic processing in a sentential context.

### 1.4. Research Aims and Hypotheses

The present study aimed to determine whether and how positive and negative moods modulate lexico-semantic processing in unbalanced Polish–English bilinguals. To test this, we instructed participants to watch positive- and negative-mood-inducing animated film clips and make semantic decisions on L1 and L2 sentences while their electroencephalographic (EEG) activity was being recorded. 

Building upon previous research, we expected to observe a classic N400 modulation by sentence meaningfulness, with more pronounced N400 amplitudes for meaningless than meaningful sentences (e.g., “These houses were transformed into country lobsters/mansions permanently.”) in both L1 and L2 [22]. We also hypothesised that while N400 amplitude would be differently modulated by positive and negative moods in L1 (i.e., N400 amplitudes elicited by meaningful sentences would be reduced in a negative relative to positive mood) [28,30], L2 processing would be less sensitive to mood manipulation. 

In addition to regulatory effects of mood on lexico-semantic processing indexed by N400 modulations [23,28,29], emerging evidence suggests that positive and negative moods also affect other language processing stages such as lexical processing (indexed by changes in N1 responses) [4] and semantic re-evaluation (indexed by changes in LPC responses) [4,23]. Therefore, to better understand the complexity of mood effects on bilingual language processing, we exploratorily analysed four additional ERP components marking its other stages: P1 (i.e., indexing perceptual processing), N1 (i.e., indexing lexical processing), N2 (i.e., indexing lexico-semantic processing), and LPC (i.e., indexing semantic integration and re-evaluation).

## 2. Materials and Methods

### 2.1. Participants

Thirty Polish–English (L1–L2) bilinguals participated in the study. Four datasets had to be excluded from the analyses due to low quality of the recorded EEG signal. As arousal may affect mood effects on language processing [5], four further datasets were excluded on the basis of exceptionally low arousal ratings (i.e., a decrease/no change in arousal post- relative to pre-experiment) to match the arousal level between the positive and negative mood conditions. The final sample thus consisted of 22 participants aged 22–32 (M_Age_ = 25.91, 95% CI (24.74, 27.08)), who were graduate students of English Studies at Adam Mickiewicz University, Poznań, engaged in an intensive English-only curriculum (the C2 level of the Common European Framework of Reference, CEFR). Due to gender-driven mood effects on language processing observed in previous research [5], only females participated in the present study. Consistent with de Groot [31], participants were classified as highly proficient unbalanced late Polish–English bilinguals who had not lived in the L2 (English) environment and had acquired their L2 in an instructional yet immersive learning context (see Table 1). All participants were in a good general affective state, reporting low degrees of depression, anxiety, and stress around the time of data collection (see Table 2). All participants had normal/corrected-to-normal vision and hearing and no neurological, mood, or language disorders. Personality-wise, participants were characterised as somewhat extraverted, emotionally stable, and highly agreeable, conscientious, and open to new experiences (see Table 2). They also reported being able to empathise with others, including fictitious characters (see Table 2) [1]. For their participation, they received a gift card of 300 PLN.

### 2.2. Linguistic Stimuli

The linguistic stimuli consisted of 90 Polish and 90 English concrete emotionally-neutral nouns (see Table 3 for details on their lexico-semantic properties) embedded in a sentence mid-position of 90 constraining sentence frames in each language. Each sentence frame was used twice, once each with a semantically congruent and incongruent critical word (e.g., “These houses were transformed into country mansions/lobsters permanently.”), which summed up to 360 unique sentences (see at https://osf.io/e3r28/ (accessed on 25 February 2022)).

All sentences were of 8–10 words (M = 9.00, 95% CI (8.88, 9.12) for both Polish and English items), declarative, emotionally neutral, highly constraining, and devoid of personal references (to avoid a self-positivity bias [47]). The critical words were presented as the seventh word of a sentence. To avoid possible processing strategies due to the fixed sentence position of the critical words, we constructed 60 Polish and 60 English filler sentences containing a semantically incongruent item as the eighth/ninth/tenth word. This yielded a final set of 480 sentences, half of which were presented during the first experimental session and half of which were presented during the second experimental session. The meaningful and meaningless sentences with the same critical word were not presented during the same experimental session. 

All the sentences were rated in a norming study on their meaningfulness (i.e., meaningfulness ratings), the probability of encountering them in everyday communicative interactions (i.e., the probability ratings), and the emotional value of each sentence frame (i.e., the valence ratings; see Table 4 for details on raters). All the ratings were analysed with a linear mixed-effects model (LMM) [48,49,50,51], using the lme4 package (Version 1.1-23) [52] for R (R Development Core Team, 2020, Vienna, Austria). Sum contrasts were applied to all categorical factors. A maximal model was first computed with a full random-effect structure, including subject- and item-related variance components for intercepts and by-subject and by-item random slopes for fixed effects [49]. When the data did not support the execution of the maximal model random structure, we reduced the model complexity to arrive at a parsimonious model [53]. To do so, we computed principal component analyses of the random structure and then kept the number of principal components that cumulatively accounted for 100% of the variance. *b* estimates and significance of fixed effects and interactions (*p*-values) were based on the Satterthwaite approximation for LMM (the lmerTest package, Version 3.1.2.) [54] for R (R Development Core Team, 2020, Vienna, Austria). Post-hoc analyses were calculated using the emmeans package (Version 1.7.0) [55] for R (R Development Core Team, 2020, Vienna, Austria).

The analysis performed on the meaningful ratings showed a fixed effect of sentence type, *b* = 4.76, SE = 0.08, *t*(69.62) = 56.93, *p* < 0.001, such that meaningful sentences were rated as more meaningful than meaningless sentences (see Table 4). There was also a fixed effect of language, *b* = 0.18, SE = 0.08, *t*(72.10) = 2.28, *p* = 0.026, whereby English sentences were rated as more meaningful than Polish sentences (see Table 4). The analysis also revealed a language × sentence type interaction, *b* = −0.39, SE = 0.16 *t*(69.93) = −2.35, *p* = 0.022. Post-hoc comparisons showed that while meaningless English relative to Polish sentences scored higher on meaningfulness, *b* = 0.37, SE = 0.13, *t*(65.60) = 2.81, *p* = 0.039, there was no such between-language difference for meaningful sentences, *b* = −0.01, SE = 0.09, *t*(84.20) = −0.16, *p* = 1.00 (see Table 4). Similarly, the analysis performed on the probability ratings yielded a fixed effect of sentence type, *b* = 4.13, SE = 0.08, *t*(96.87) = 48.46, *p* < 0.001, such that meaningful sentences were rated as more probable to be encountered in everyday interactions compared to meaningless sentences (see Table 4). There was also a fixed effect of language, *b* = 0.40, SE = 0.10, *t*(84.38) = 4.13, *p* < 0.001, whereby English sentences were rated as being more probable to be encountered in everyday interactions than Polish sentences (see Table 4). The analysis also revealed a language × sentence type interaction, *b* = 0.83, SE = 0.17, *t*(97.71) = 4.93, *p* < 0.001. Post-hoc comparisons showed that while English meaningful sentences scores higher on predictability, *b* = 0.81, SE = 0.16, *t*(73.70) = 5.10, *p* < 0.001, there was no such between-language difference for meaningless sentences, *b* = −0.02, SE = 0.09, *t*(190.60) = −0.24, *p* = 1.00 (see Table 4). Finally, there was no between-language difference for the valence ratings, *b* = 0.03, SE = 0.07, *t*(152.21) = 0.41, *p* = 0.686 (see Table 5).

### 2.3. Mood-Inducing Stimuli

To induce a positive or negative mood in our participants, we employed 28 affectively evocative animated film clips of 90 s each adapted from Naranowicz et al. [5]. The clips contained no spoken or written words to avoid a possible priming effect of language. In total, participants watched 21 min of such an audio–video material during each experimental session. Each clip was rated in a norming study (see Table 4 for details on raters) on mood valence (1—the film evokes strongly negative emotions, 7—the film evokes strongly positive emotions) and arousal (1—the film makes me feel completely unaroused, 7—the film makes me feel highly aroused). Fourteen clips with the highest and lowest valence were then used as the ones inducing a positive mood (M_Valence_ = 5.34, 95% CI (5.17, 5.52); M_Arousal_ = 3.62, 95% CI (3.06, 4.17)) and a negative mood (M_Valence_ = 1.97, 95% CI (1.78, 2.16); M_Arousal_ = 4.27, 95% CI (3.94, 4.59)), respectively. The ratings were analysed with a linear mixed-effects model (LMM) [48,49,50,51], using the lme4 package (version 1.1–23) [52] for R (R Development Core Team, 2020, Vienna, Austria). The analysis of the mood valence ratings yielded a fixed effect of film type, *b* = −3.37, SE = 0.18, *t*(47.30) = −18.92, *p* < 0.001, whereby the film clips selected to induce a positive mood were rated higher in valence than those selected to induce a negative mood (M_PositiveMood_ = 5.34, 95% CI (5.17, 5.52); M_NegativeMood_ = 1.97, 95% CI (1.78, 2.16)), *t*(20.98) = −24.94, *p* < 0.001. Then, the analysis of the arousal ratings showed no difference between the two film types in terms of how arousing they were (M_PositiveMood_ = 3.62, 95% CI (3.06, 4.17); M_NegativeMood_ = 4.27, 95% CI (3.94, 4.59)), *b* = 0.65, SE = 0.41, *t*(38.40) = 1.57, *p* = 0.124.

### 2.4. Procedure

The procedure applied in the experiment was approved by the Ethics Committee for Research Involving Human Participants at Adam Mickiewicz University, Poznań. The experiment was conducted at the Neuroscience of Language Laboratory (Faculty of English, Adam Mickiewicz University, Poznań). Potential participants were initially screened by means of an online version of DASS-21 [39], the PANAS test [34], and an additional medical history questionnaire.

Each of the two sessions (conducted one week apart) involved either a positive or negative mood induction (counterbalanced order). Participants were seated in a dimly lit and quiet booth, 75 cm away from a LED monitor with a screen resolution of 1280 × 1024 pixels. All remaining questionnaires (see Table 1 and Table 2) were administered during EEG cap preparation to build participants’ linguistic and socio-biographical profiles. E-prime 3.0 software was used to present the stimuli and collect the behavioural data, and BrainVision Recorder 1.23 (Gilching, Germany) was used to collect the EEG data.

**Figure 1:**
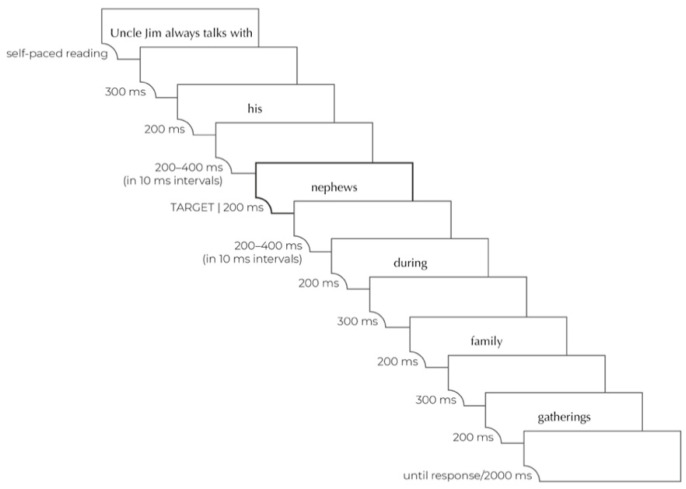
Time sequence of stimulus presentation.

Participants were asked to rate their mood prior to and post mood manipulation based on the mood valence and arousal ratings and the Polish version of PANAS [34]. Participants first watched three film clips to induce the targeted mood and were instructed to put themselves in the targeted mood [56], to imagine that they were one of the protagonists, and to sympathise with other characters [57]. Participants performed a semantic decision task, wherein they decided whether a sentence was meaningless or meaningful by pressing corresponding keys (counterbalanced order and key designation). Another film clip was presented every 20 sentences (counterbalanced order) to sustain the targeted mood. Participants completed one Polish and one English block within each session (counterbalanced order), each comprising 45 meaningful, 45 meaningless, and 30 filler (meaningless) sentences. The time sequence of stimulus presentation is provided in Figure 1.

### 2.5. EEG Data Recording

EEG signals were recorded from 64 active actiCAP slim electrodes (Brain Products), placed at the standard extended 10–20 positions with the ground placed at Fpz. The bipolar electrodes monitoring vertical (vEOG) and horizontal (hEOG) eye movements were placed above and below the left eye and next to the outer rims of both eyes, respectively. EEG signal was recorded using BrainVision actiCHamp amplifiers (Brain Products, Gilching, Germany), sampled at 500 Hz/channel, and referenced to the Fz electrode. Impedances were kept below 10 kΩ for each electrode. ERPs were time-locked to the onset of the seventh (critical) word of each sentence.

### 2.6. Behavioural Data Analysis

All analyses were performed in the R environment (Version 4.0; R Development Core Team, 2020, Vienna, Austria). Participants rated their mood on 7-point mood valence and arousal scales, similarly to the norming study (see the Mood-Inducing Stimuli section for details), and a Polish version of PANAS [34], employing a 5-point Likert scale (1 —very slightly or not at all, 5 —extremely) with 10 positive adjectives (i.e., positive affect scores) and 10 negative adjectives (i.e., negative affect scores; see Table 2). Then, the positive and negative affect scores were summed separately and presented as a proportion of the summed positive to negative affect scores. All adjectives had feminine forms. Mood ratings were analysed using linear mixed-effects modelling (LMM), with the lme4 package (Version 1.1-23) for R (R Development Core Team, 2020, Vienna, Austria) [52] (see the Linguistic Stimuli section for details), on the basis of a 2 (time of testing: pre-experiment vs. post-experiment) × 2 (film type: positive vs. negative) within-subject design. To ensure the effectiveness of our mood manipulation, we compared mood valence, arousal, and PANAS ratings post- relative to pre-experiment separately in each mood condition as planned comparisons, predicting an increase/no change in mood ratings in the positive mood condition and a decrease in the negative mood condition.

Response accuracy data were analysed with a generalised linear mixed-effects model (GLMM; i.e., logistic regression) [48,49,50,51], using the lme4 package (Version 1.1-23) [52] for R (R Development Core Team, 2020, Vienna, Austria), on the basis of a 2 (language: Polish vs. English) × 2 (mood: positive vs. negative) × 2 (sentence type: meaningful vs. meaningless) within-subject design (see the Linguistic Stimuli section for details). 

### 2.7. Electrophysiological Data Analysis

We analysed two ERP components previously reported to be modulated by semantic anomalies [58], language of operation [13], and mood [23]: the N400 (300–500 ms) and LPC (600–800 ms). Both components were analysed over 9 electrodes: FC1, FCz, FC2 (fronto-central), C1, Cz, C2 (central), CP1, CPz, and CP2 (centro-parietal) [12,13]. Moreover, as Kissler and Bromberek-Dyzman [4] observed early modulatory effects of mood on bilingual word processing, we exploratorily analysed the P1 (70–130 ms), N1 (170–230 ms), and N2 (250–350 ms) components previously linked to the pre-lexical (P1), lexical (N1), and lexico-semantic (N2) stages of language processing, whose time windows were selected based on visual inspection of the averaged ERPs and of electrodes at maximal peak amplitude. P1 and N1 were analysed over 4 electrodes: PO7, PO8 (parieto-occipital), P7, and P8 (parietal), whereas N2 was analysed over 6 electrodes: F1, Fz, F2 (frontal), FC1, FCz, and FC2 (fronto-central).

BrainVision Analyzer 2.1 software (Brain Products, Gilching, Germany) was used to analyse the data offline. Continuous EEG data were re-referenced to the common average reference [24,59], filtered offline (Butterworth zero-phase filter) with a high-pass cut-off set at 0.1 Hz (slope 24 dB/octave) and a low-pass cut-off set at 20 Hz (slope 24 dB/octave) and then epoched from 200 ms before critical word onset to 1500 ms afterwards. Then, the data were baseline-corrected relative to signal between −200 and 0 ms before stimulus onset and edited for artifacts (i.e., rejecting trials with flat lines at 0 μV and rejecting trials with voltage differences higher than 100 μV or voltage steps higher than 50 μV). Ocular artifacts were corrected using the ocular artifact regression method proposed by Gratton and Coles [60].

Mean P1, N1, and N2 amplitudes were analysed using RM ANOVAs on the basis of a 2 (language: Polish vs. English) × 2 (mood: positive vs. negative) × 2 (sentence type: meaningful vs. meaningless) within-subject design. Mean N400 and LPC amplitudes were analysed using RM ANOVAs on the basis of a 2 (language: Polish vs. English) × 2 (mood: positive vs. negative) × 2 (sentence type: meaningful vs. meaningless) × 3 (laterality: left-lateral vs. medial vs. right-lateral) × 3 (electrode position: anterior vs. central vs. posterior) within-subject design. In all analyses, pairwise comparisons were Bonferroni corrected. Greenhouse–Geisser correction was applied when the sphericity assumption was violated.

Moreover, Pearson correlation coefficients (*r*) were calculated to further explore whether there was a linear relationship between the observed effects and participants’ mood ratings along with their linguistic (see Table 1) and personality-based characteristics (see Table 2). All R scripts and full model specifications can be found at https://osf.io/e3r28/ (accessed on 25 February 2022).

## 3. Results

### 3.1. Self-Report Data: Mood Ratings

**Figure 2:**
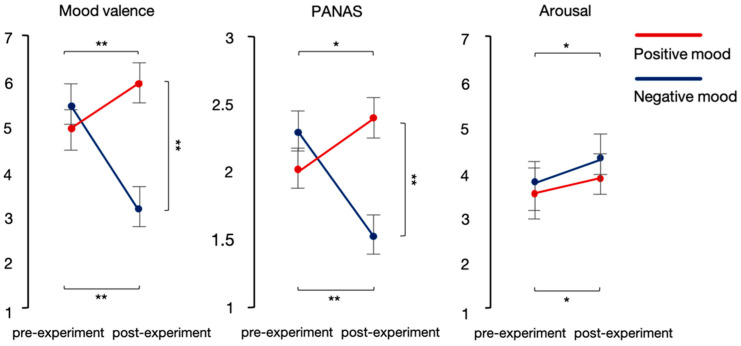
Mood ratings for the mood valence scale (**left**), the PANAS (**middle**), and the arousal scale (**right**) with CI of 95% (** *p* < 0.001, * *p* < 0.01).

The analysis performed on the mood valence ratings showed fixed effects of both film type, *b* = 1.21, SE = 0.16, *t*(63) = 7.68, *p* < 0.001, and testing time, *b* = −0.89, SE = 0.16, *t*(63) = −5.65, *p* < 0.001, along with a film type × testing time interaction, *b* = 3.41, SE = 0.31, *t*(63) = 10.86, *p* < 0.001. As expected, planned comparisons showed an increase in valence ratings in post- compared to pre-experiment mood ratings in the positive mood condition, *b* = 0.82, SE = 0.22, *t*(63) = 3.69, *p* = 0.003, and a decrease in the negative mood condition, *b* = −2.59, SE = 0.22, *t*(63) = −11.68, *p* < 0.001 (see Figure 2 and Table 6). Similarly, the analysis of the PANAS ratings revealed fixed effects of both film type, *b* = 0.39, SE = 0.08, *t*(63) = 4.97, *p* < 0.001, and testing time, *b* = −0.31, SE = 0.08, *t*(63) = −3.86, *p* < 0.001, along with a film type × testing time interaction, *b* = 1.23, SE = 0.16, *t*(63) = 7.75, *p* < 0.001. Planned comparisons showed an increase in the PANAS ratings between pre- and post-experiment in the positive mood condition, *b* = 0.31, SE = 0.11, *t*(63) = 2.75, *p* = 0.047, and a decrease in the negative mood condition, *b* = −0.70, SE = 0.11, *t*(63) = −6.25, *p* < 0.001 (see Figure 2 and Table 6). The analysis of the arousal ratings showed only one main effect of testing time, *b* = 0.50, SE = 0.20, *t*(63) = 2.55, *p* = 0.013, such that participants felt more emotionally aroused after the experiment than before it began, regardless of mood type (see Figure 2 and Table 6).

Additional correlational analyses revealed that mood valence ratings correlated positively with the Interpersonal Reactivity Index [42] empathetic concern scores in a positive mood, *r* = 0.43, 95% CI (0.02, 0.72), *t*(21) = 2.18, *p* = 0.041, as well as negatively with the DASS-21 [39] depression scores in a negative mood, *r* = −0.41, 95% CI (−0.71, −0.01), *t*(21) = −2.25, *p* = 0.049. Then, the analyses also indicated that participants’ arousal level in a negative mood correlated negatively with their familiarity with the mood-inducing film clips, *r* = −0.43, 95% CI (−0.72, −0.02), *t*(21) = −2.19, *p* = 0.040, as well as positively with the Interpersonal Reactivity Index [42] empathetic concern scores, *r* = −0.49, 95% CI (0.10, 0.75), *t*(21) = 2.59, *p* = 0.017.

### 3.2. Behavioural Data: Response Accuracy

The analysis performed on response accuracy showed a fixed effect of language, *b* = −0.59, SE = 0.25, *z* = −2.32, *p* = 0.020, whereby Polish (L1) sentences (M = 97.44%, 95% CI (90.69, 100.00)) were responded to with greater accuracy than English (L2) sentences (M = 95.80%, 95% CI (87.22, 100.00)). The analysis also yielded a fixed effect of sentence type, *b* = −0.77, SE = 0.32, *z* = −2.41, *p* = 0.016, such that meaningless sentences (M = 97.16%, 95% CI (90.06, 100.00)) were responded to with greater accuracy than meaningful sentences (M = 96.14%, 95% CI (87.90, 100.00)).

The analysis also revealed a mood × sentence type interaction, *b* = 1.13, SE = 0.50, *z* = 2.28, *p* = 0.023. Post-hoc comparisons showed that while meaningless relative to meaningful sentences were responded to with greater accuracy in the negative mood, (M_Meaningful_ = 95.68%, 95% CI (86.99, 100.00); M_Meaningless_ = 97.19%, 95% CI (90.11, 100.00)), *b* = −1.33, SE = .46, *z* = −2.92, *p* = 0.021, there was no such between-sentence-type difference in the positive mood (M_Meaningful_ = 96.59%, 95% CI (88.82, 100.00); M_Meaningless_ = 97.14%, 95% CI (90.01, 100.00)), *b* = −0.20, SE = 0.34 *z* = −0.58, *p* = 0.99. All the remaining differences in response accuracy were non-significant, *p*s > 0.05.

### 3.3. Electrophysiological Data

#### 3.3.1. P1 Time Window (70–130 ms)

The RM ANOVA performed within the P1 time window (70–130 ms) showed a main effect of language, *F*(1, 21) = 10.36, *p* = 0.004, η_p_^2^ = 0.330, whereby P1 amplitudes were more pronounced in response to English (L2) relative to Polish (L1) sentences. There was also a main effect of mood, *F*(1, 21) = 4.51, *p* = 0.046, η_p_^2^ = 0.177, such that P1 amplitudes were larger in the positive as compared to the negative mood condition. All the remaining differences in P1 mean amplitudes were non-significant, *p*s > 0.05.

**Figure 3:**
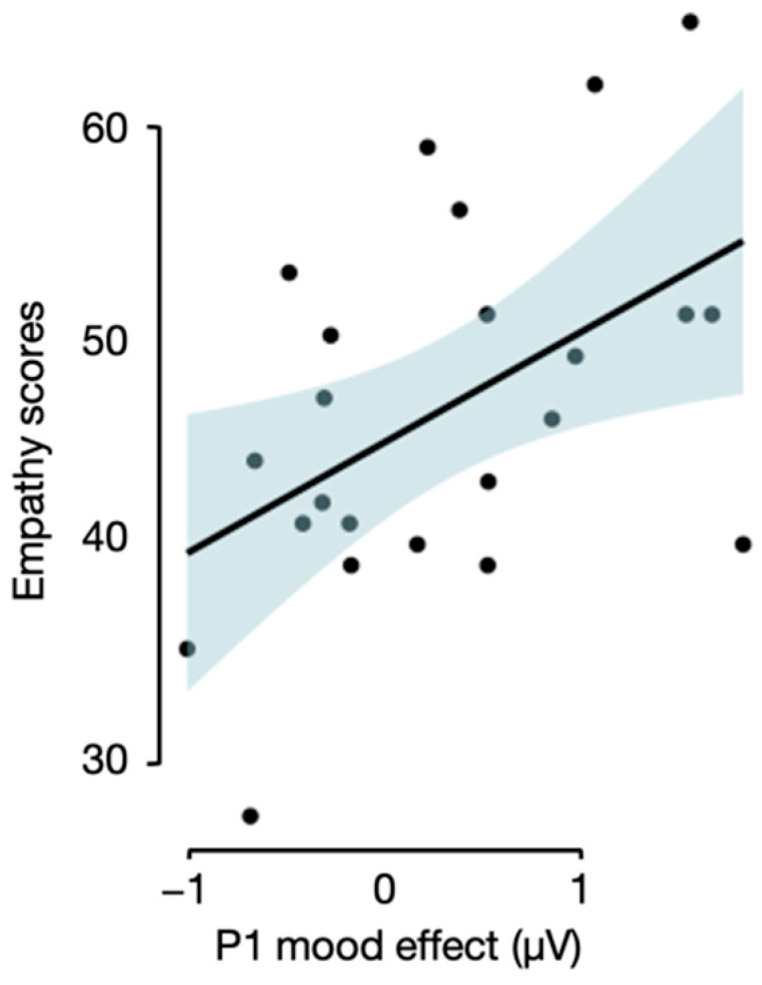
A correlation plot depicting the relationship between the P1 mood effect and participants’ empathy level.

Moreover, a correlational analysis indicated that the P1 mood effect (i.e., the difference in P1 amplitudes between a positive and negative mood) correlated positively with the Empathy Quotient scores [38], *r* = 0.49, 95% CI (0.10, 0.75), *t*(21) = 2.56, *p* = 0.018 (see Figure 3).

#### 3.3.2. N1 Time Window (170–230 ms)

The RM *ANOVA* performed within the N1 time window (170–230 ms) showed a main effect of language, *F*(1, 21) = 10.04, *p* = 0.005, η_p_^2^ = 0.332, whereby English (L2) sentences elicited a more pronounced N1 response compared to Polish (L1) sentences. 

We also found a language × sentence type interaction, *F*(1, 21) = 5.49, *p* = 0.029, η_p_^2^ = 0.207. Post-hoc paired sample *t*-tests showed that while English (L2) meaningless sentences elicited a more pronounced N1 response compared to Polish (L1) meaningless sentences, *t*(21) = −4.44, *p* < 0.001, there was no such between-language difference for meaningful sentences, *t*(21) = −1.69, *p* = 0.105. 

**Figure 4:**
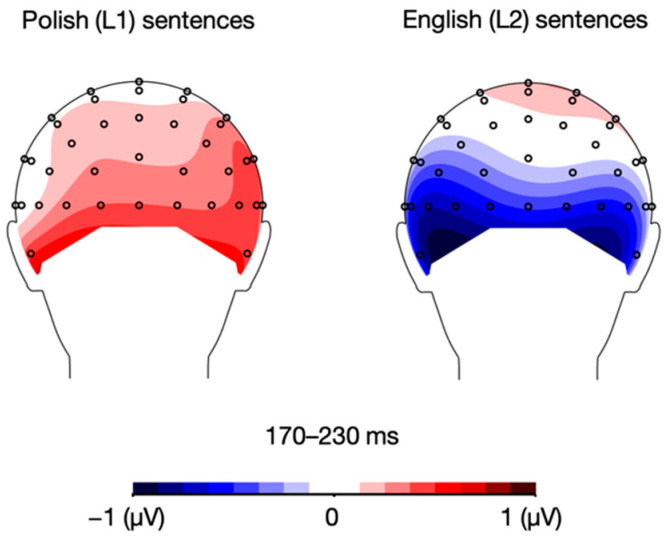
Topographic distribution of the difference between ERP amplitudes in the positive and negative mood conditions for Polish (L1) and English (L2) sentences in the 170–230 ms time window.

**Figure 5:**
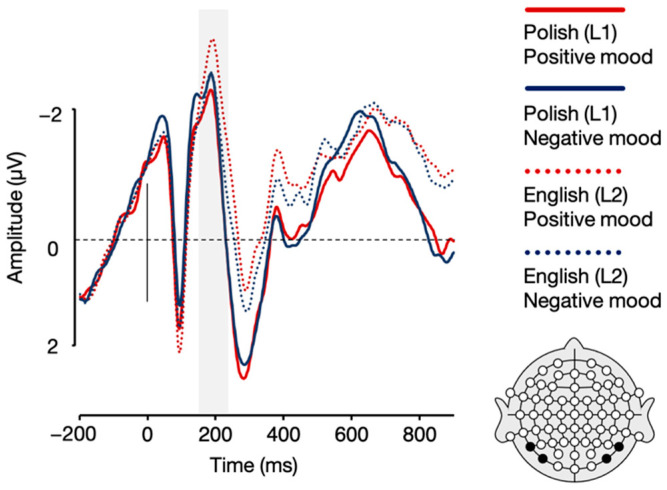
Grand averages for Polish (L1) and English (L2) sentences in the positive and negative mood conditions over parietal (P7, P8) and parieto-occipital (PO7, PO8) electrodes.

Crucially, the analysis also showed a language × mood interaction, *F*(1, 21) = 8.11, *p* = 0.010, η_p_^2^ = 0.279. Post-hoc paired sample *t*-tests showed that while English (L2) sentences elicited a more pronounced N1 response in the positive compared to negative mood condition, *t*(21) = −2.66, *p* = 0.015, the analysis showed no such between-mood difference for Polish (L1) sentences, *t*(21) = 1.39, *p* = 0.180 (see Figure 4 and Figure 5). All the remaining differences in N1 mean amplitudes were non-significant, *p*s > 0.05.

#### 3.3.3. N2 Time Window (250–350 ms)

The RM *ANOVA* performed within the N2 time frame (250–350 ms) showed a main effect of sentence type, *F*(1, 21) = 25.62, *p* < 0.001, η_p_^2^ = 0.550, whereby meaningless sentences elicited larger N2 amplitudes than meaningful sentences. There was also a main effect of language, *F*(1, 21) = 46.07, *p* < 0.001, η_p_^2^ = 0.687, such that Polish (L1) sentences elicited more pronounced N2 amplitudes than English (L2) sentences. The analysis also revealed a main effect of mood, *F*(1, 21) = 5.47, *p* = 0.029, η_p_^2^ = 0.207, whereby the N2 amplitudes were more pronounced in the positive than the negative mood condition.

**Figure 6:**
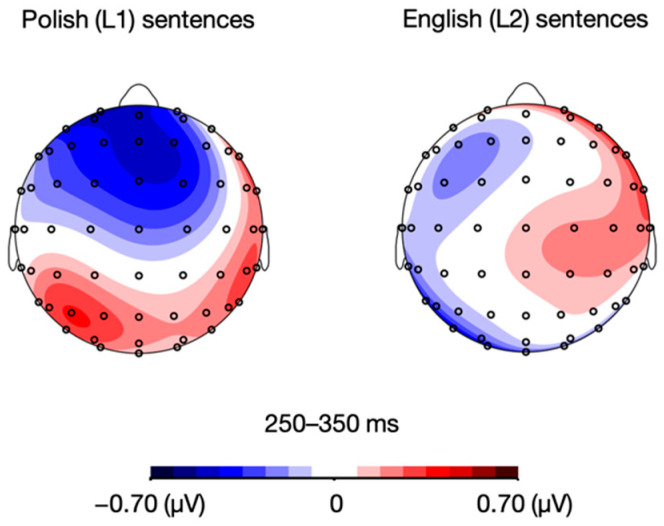
Topographic distribution of the ERP amplitude difference between positive and negative mood conditions for Polish (L1) and English (L2) sentences in the 250–350 ms time window.

**Figure 7:**
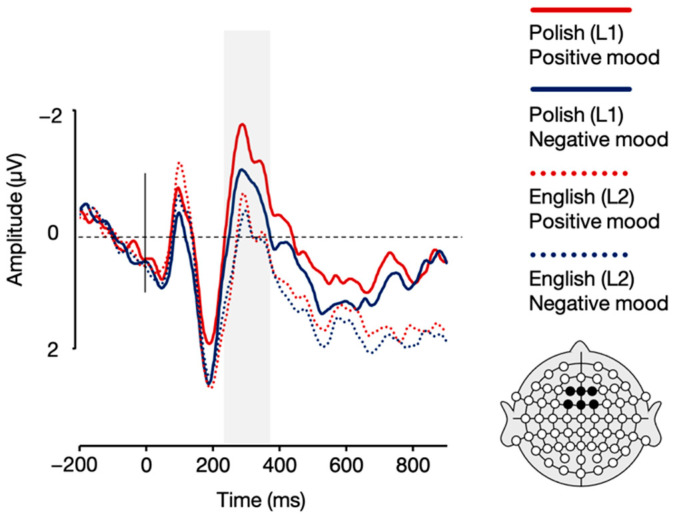
Grand average ERPs for Polish (L1) and English (L2) sentences in the positive and negative mood conditions over frontal (F1, Fz, F2) and fronto-central (FC1, FCz, FC2) electrodes.

Importantly, the analysis also revealed a language × mood interaction, *F*(1, 21) = 4.96, *p* = 0.037, η_p_^2^ = 0.191. Post-hoc paired sample *t*-tests showed that while Polish (L1) sentences elicited a more pronounced N2 response in the positive compared to the negative mood condition, *t*(21) = –2.89, *p* = 0.009, whereas no such between-mood significant difference was found for English (L2) sentences, *t*(21) = –0.59, *p* = 0.561 (see Figure 6 and Figure 7). All the remaining differences in N2 mean amplitudes were non-significant, *p*s > 0.05.

#### 3.3.4. N400 Time Window (300–500 ms)

The RM *ANOVA* performed within the N400 time frame (300–500 ms) showed a main effect of sentence type, *F*(1, 21) = 39.28, *p* < 0.001, η_p_^2^ = 0.582, such that the N400 amplitudes were more pronounced in response to meaningless than meaningful sentences.

The analysis also yielded a mood × language × sentence type × electrode position interaction, *F*(2, 42) = 3.57, *p* = 0.048, η_p_^2^ = 0.145, ε = 0.769. To deconstruct it, we conducted language × sentence type × electrode position post-hoc ANOVAs separately for each mood. The analyses showed a significant main effect of sentence type, with more robust N400 amplitudes for meaningless relative to meaningful sentences in both Polish (L1), *F*(1, 21) = 15.72, *p* < 0.001, η_p_^2^= 0.428, and English, *F*(1, 21) = 29.36, *p* < 0.001, η_p_^2^ = 0.583.

Then, unlike for the negative mood condition, the analyses for the positive mood condition showed a language × sentence type × electrode position interaction, *F*(2, 42) = 6.41, *p* = 0.012, η_p_^2^ = 0.234, ε = 0.642. It was further deconstructed via language × sentence type post-hoc *ANOVA*s conducted separately for fronto-central (FC1, FCz, and FC2), central (C1, Cz, and C2), and centro-parietal (CP1, CPz, and CP2) electrodes. We observed the main effect of sentence type over fronto-central, *F*(1, 21) = 11.35, *p* = 0.003, η_p_^2^ = 0.351, central, *F*(1, 21) = 16.24, *p* < 0.001, η_p_^2^ = 0.436, and centro-parietal electrodes, *F*(1, 21) = 10.30, *p* = 0.004, η_p_^2^ = 0.329, whereby meaningless sentences evoked more pronounced N400 amplitudes compared to meaningful utterances in the positive mood condition.

**Figure 8:**
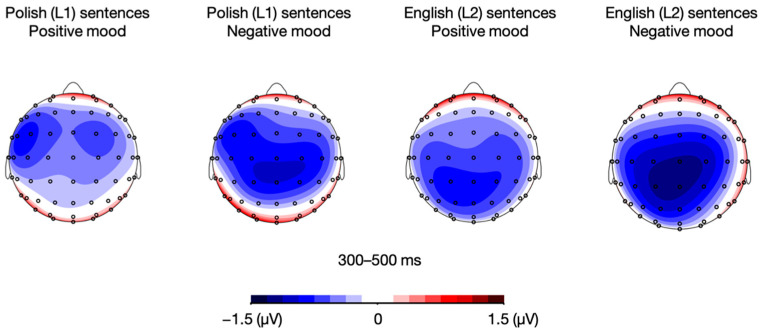
Topographic distribution of the difference between ERP amplitudes in the meaningful and meaningless conditions for Polish (L1) and English (L2) sentences in the positive and negative mood conditions in the 300–500 ms time window.

**Figure 9:**
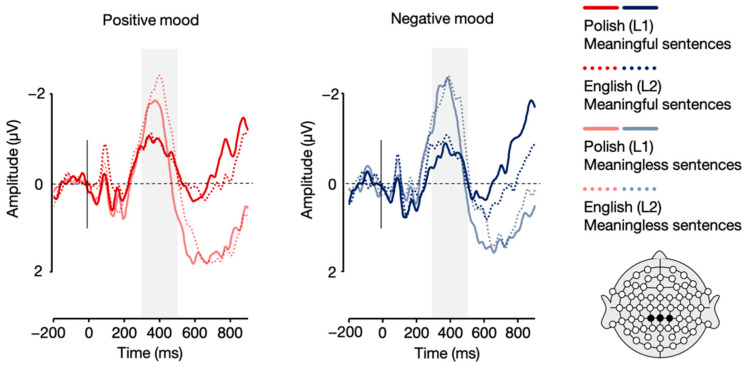
Grand averages for Polish (L1) and English (L2) meaningful and meaningless sentences in the positive and negative mood conditions over centro-parietal (CP1, CPz, CP2) electrodes.

Importantly, the analyses for the positive mood condition also revealed a language × sentence type interaction over centro-parietal electrodes, *F*(1, 21) = 6.67, *p* = 0.017, η_p_^2^ = 0.241. Post-hoc paired sample *t*-tests showed that in a positive mood, while English (L2) meaningless sentences evoked higher N400 amplitudes relative to meaningful sentences, *t*(21) = 3.70, *p* = 0.001, there was no such between-sentence-type difference for Polish (L1) sentences, *t*(21) = 1.46, *p* = 0.160. Additionally, the post-hoc *t*-tests also revealed attenuated N400 amplitudes for Polish (L1) compared to English (L2) meaningless sentences in the positive mood condition, *t*(21) = −2.40, *p* = 0.026, with no such between-language difference for meaningful sentences, *t*(21) = 0.10, *p* = 0.919 (see Figure 8 and Figure 9). All the remaining differences in N400 mean amplitudes were non-significant, *p*s > 0.05.

#### 3.3.5. LPC Time Window (600–800 ms)

The RM *ANOVA* performed within the LPC time frame (600–800 ms) showed a main effect of sentence type, *F*(1, 21) = 27.68, *p* < 0.001, η_p_^2^ = 0.569, such that meaningless sentences elicited increased positivity relative to meaningful sentences. The analysis also yielded a main effect of language, *F*(1, 21) = 4.34, *p* = 0.050, η_p_^2^ = 0.171, whereby English (L2) sentences elicited a more pronounced LPC response than Polish (L1) sentences.

We also found a language × sentence type interaction, *F*(1, 21) = 5.49, *p* = 0.029, η_p_^2^ = 0.207. Post-hoc paired sample *t*-tests showed that while English (L2) meaningful sentences elicited a more pronounced LPC response compared to Polish (L1) meaningful sentences, *t*(21) = 2.99, *p* = 0.007, there was no such between-language difference for meaningless sentences, *t*(21) = 0.87, *p* = 0.395.

The analyses also showed a mood × language × sentence type interaction, *F*(1, 21) = 4.98, *p* = 0.037, η_p_^2^ = 0.192. To deconstruct it, we performed language × sentence type post-hoc ANOVAs separately for each mood. The analyses showed a significant main effect of sentence type in both the positive mood condition, *F*(1, 21) = 32.62, *p* < 0.001, η_p_^2^= 0.608, and the negative mood condition, *F*(1, 21) = 29.36, *p* < 0.001, η_p_^2^ = 0.583.

**Figure 10:**
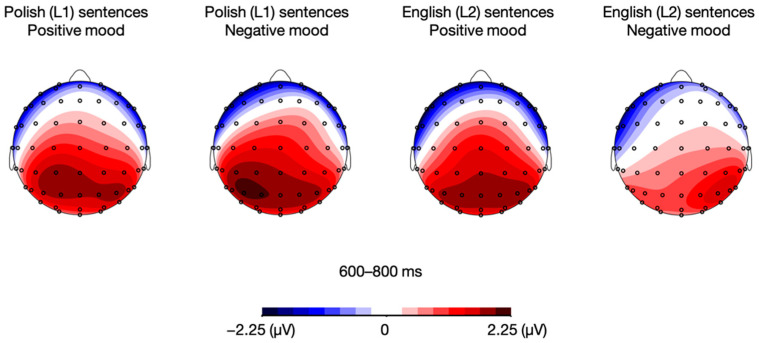
Topographic distribution of the difference between ERP amplitudes in the meaningful and meaningless conditions for Polish (L1) and English (L2) sentences in the positive and negative mood conditions in the 600–800 ms time window.

**Figure 11:**
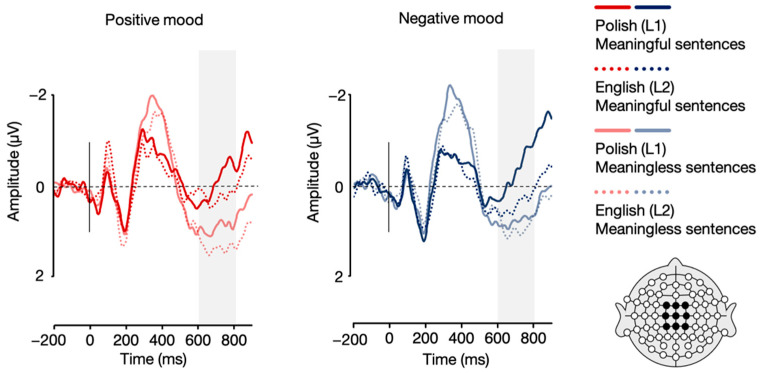
Grand averages for Polish (L1) and English (L2) meaningful and meaningless sentences in the positive and negative mood conditions over fronto-central (FC1, FCz, FC2), central (C1, Cz, C2), and centro-parietal (CP1, CPz, CP2) electrodes.

Then, unlike in the positive mood condition, the analysis for the negative mood condition yielded a language × sentence type interaction, *F*(1, 21) = 6.78, *p* = 0.017, η_p_^2^ = 0.244. Post-hoc paired sample *t*-tests performed for the negative mood condition showed between-language differences for meaningful sentences, with an attenuated LPC response to Polish (L1) relative to English (L2) meaningful utterances, *t*(21) = 3.37, *p* = 0.003. However, such a between-language difference was not found for meaningless sentences, *t*(21) = −0.07, *p* = 0.948 (see Figure 10 and Figure 11). All the remaining differences in LPC mean that amplitudes were non-significant, *p*s > 0.05.

## 4. Discussion

The present study investigated how positive and negative moods modulate lexico-semantic processing (as indexed by N400 responses) in L1 and L2 of unbalanced Polish–English (L1–L2) bilinguals. Besides a classic N400 modulation by meaningfulness [22], we expected to observe an N400 effect of meaningfulness to be differently modulated by mood in L1 and L2, as suggested by recent evidence pointing to the activation of narrowed and detailed-oriented cognitive processing in a negative mood in L1 [28], reduced sensitivity to emotional content in L2 compared to L1 [6,7,8,9,10,11,12,13,14,15,61,62], and more effective emotion regulation processes in bilinguals operating in L2 relative to L1 [18]. In order to thoroughly analyse mood effects on bilingual language processing, we also exploratorily analysed other language-related ERP components: P1 (i.e., a marker of pre-lexical perceptual processing), N1 (i.e., a marker of lexical processing), N2 (i.e., a marker of lexico-semantic processing), and LPC (i.e., a marker of semantic re-analysis and integration).

### 4.1. Perceptual Processing: P1

The P1 component has been considered an index of perceptual processing of stimuli (including linguistic ones), reflecting early sensory processing in the visual modality that are modulated by attention [25]. Consequently, P1 has also been employed to investigate mood effects on attention (see [63] for a review). Here, we observed larger P1 amplitudes in a positive compared to a negative mood, consistent with research pointing to increased attentional focus in a positive relative to a negative mood [63,64,65]. For instance, in a flanker task (i.e., differentiating between strings with identical and incompatible letters), Moriya and Nittono [63] observed a larger probe-evoked P1 response in a positive relative to a negative mood induced by affective pictures. They suggested that the broadened attentional scope in a positive mood reflects the brain activity in the extrastriate visual cortices, which are responsible for early visual attention [66]. Interestingly, our correlational analysis also pointed to the strength of the P1 mood effect increasing proportionally to empathy level, suggesting that an increase in empathy may lead to an increase in mood’s effects on perception and attention regulation.

We also found more robust P1 amplitudes in response to L2 than L1. Previous research has shown that the P1 can also be modulated by participants’ arousal level. For instance, Vogel and Luck [67] manipulated the difficulty of a perceptual task to increase participants’ physiological arousal and reported larger P1 amplitudes in highly compared to moderately aroused participants. A similarly difficulty-driven mechanism might have been elicited in our participants, and thus modulations within the P1 response might reflect increased difficulty (and hence arousal) due to the performance of a cognitive task testing our participants’ comprehension of their less proficient and less dominant language. Such an interpretation is also in line with previous research that has typically pointed to less automatic and more cognitively taxing mechanisms in L2 [68].

### 4.2. Lexical Processing: N1

The visual N1 component is typically responsive to lexical attributes of words (e.g., word frequency) in word recognition tasks and is thus interpreted as an index of lexical processing (see [69] for a review). Here, we observed larger N1 responses to English (L2) than Polish (L1) words, and this effect was further modulated by mood. N1 amplitudes evoked by words in L2 sentences were reduced in a negative relative to a positive mood, and between-mood difference was not significant in L1. Our results could therefore be explained by lexical processing being comparably easy in both moods in L1, with a negative mood facilitating it in L2. Such an interpretation is consistent with research demonstrating that a negative mood can prompt a more accommodative processing mode, thus improving deception [70] and linguistic ambiguity detection [71]. Research has also shown that a negative mood can trigger behavioural, cognitive, and motivational strategies to cope with a demanding situation, activating analytic problem solving as a neural response to a potential threat [72,73]. Therefore, despite leading to unpleasant experiences, a negative mood may increase engagement in the stimuli and motivation for deeper processing when operating in L2, which results in a more effective lexical search.

Similarly, Kissler and Bromberek-Dyzman [4] found an enhanced left-lateralised N1 response to L1 words in a positive compared to negative mood, with no between-mood differences in L2. Following Schindler et al. [26], they proposed that mood may be treated as a relevant social communicative context for early word processing. Though we observed the N1 modulation by mood in the present study, its direction was inconsistent with the one observed by Kissler and Bromberek-Dyzman [4], which may be accounted for by the following differences in experimental procedures: while Kissler and Bromberek-Dyzman [4] used decontextualised positive, negative, and neutral words and asked participants to perform an emotive decision task (i.e., decide if a word is positive, negative, or neutral), our participants read neutral context-rich sentences and performed a semantic decision task. Additionally, judging by the LexTALE [32] results, the German–English bilinguals tested in the study conducted by Kissler and Bromberek-Dyzman [4] were less proficient in their L2 (English; M_LexTALE_ = 69.5%; the B2 level of CEFR) compared to our participants (M_LexTALE_ = 91.3%; the C1/C2 level of CEFR), which suggests that L2 proficiency may be another factor influencing early mood effects on language. Therefore, to provide further insights into the role of mood in lexical processing, future research could employ linguistic stimuli presented in a richer context and focus on bilinguals’ L2 proficiency.

Additionally, N1 amplitude was increased for meaningless compared to meaningful sentences in L2. As N1 is also sensitive to systematic patterns (e.g., stimulus repetition) [24], the observed effect seems to suggest that participants implicitly anticipated an aberrant word to be presented as the seventh word, despite our having included filler sentences. As in the case of P1, such repetition effect may relate to greater cognitive demands, consistent with lower interconnectivity between lexical and semantic representations in L2 [74].

### 4.3. Lexico-Semantic and Semantic Processing: N2 and N400

Similarly to N1, N2 modulations have been linked to lexical processing [74,75], particularly to inhibitory processes (indexing, e.g., conflict resolution) activated during the selection of an appropriate lexical item [76]. L2 research has also shown that N2 responses can reflect lexical processing somewhat overlapping with early lexico-semantic processing [77]. Here, we observed larger N2 amplitudes for meaningless compared to meaningful sentences, suggesting that semantic information might have been accessed early in the processing stream, possibly due to anticipation. Consistent with Proverbio et al.’s [69] findings, such an effect may result from the activation of early anticipatory processes prompted by the highly constraining sentential context, especially given that the N2 modulation appeared to carry over to the N400 time window.

We also found larger N2 amplitudes for Polish (L1) than English (L2) sentences, an effect in the opposite direction to the one observed in the P1 and N1 time windows. This could reflect less automatic activation of lexical-level representations in L2, given that the subjective frequency of L2 items is lower than that of L1 items in unbalanced bilinguals [78], in turn affecting levels of activation in the semantic network (see the spreading activation model [79]). Such an effect also discards the idea that L1 may have required more cognitive resources during this intermediate stage of language processing, potentially due to greater activation of lexical-level representations in the dominant language (i.e., L1).

As in the N1 window, we found larger N2 amplitudes in a positive compared to a negative mood, suggesting that mood effects continue to affect L1 and L2 processing in the window of lexico-semantic processing. Crucially, we also observed an attenuated N2 response to Polish (L1) sentences in a negative compared to positive mood, with no between-mood difference for English (L2) sentences. Interestingly, this mood effect on language during lexico-semantic processing appears to be a mirror reflection of the one observed during lexical processing (i.e., in the N1 range). Our interpretation of this reversal is that a negative mood may activate detail-oriented processing when a given language requires more cognitive resources, e.g., L2 during lexical processing (modulating N1) and L1 during early lexico-semantic processing (modulating N2).

We also observed modulations within the N400 time frame. N400 has been associated with lexico-semantic processing, as this component is sensitive to semantic anomalies of different types [21,22]. Here, while N400 amplitudes were more pronounced in response to English (L2) meaningless relative to meaningful sentences in both a positive and negative mood, such an effect occurred only in a negative mood for L1 processing (note that the interaction between language and sentence time found in the N1 time window (170–230 ms) most likely disappeared in the N2 time window (250–350 ms) due to systematic stimulus repetition, suggesting that effects occurring in later time windows were not carry-over effects of earlier differences). First, an attenuation of the N400 response to Polish (L1) meaningless relative to meaningful sentences in a positive mood is consistent with previous studies pointing to facilitatory effects of a positive mood on lexico-semantic processing [23,28,29,80,81]. For instance, Wang et al. [80] explored how positive and negative moods affect the processing of question–answer pairs, manipulating their semantic congruity (i.e., whether critical words were semantically congruent with the question context) and task relevance (i.e., whether critical words were relevant to questions or not). They observed that while incongruent relative to congruent words elicited larger N400 amplitudes regardless of task relevance in a negative mood, such an N400 congruity effect was observed only for task-relevant words in a positive mood. They proposed that while language users in a positive mood seem to allocate their attentional resources to the most relevant contextual information, a negative mood may trigger non-selective and analytical information processing, directing equal attention to semantic relations among all words, regardless of whether they are critical to a given context or not. Moreover, given the functional interpretation of N400 modulations in language processing (see [82] for a review), our results may relate to a positive mood requiring fewer cognitive resources than a negative mood when information is being retrieved from long-term memory during sentence comprehension. Such an interpretation is also consistent with previous evidence supporting the affect-as-information hypothesis [83], whereby positive and negative moods are thought to promote qualitatively different information processing styles. A positive mood is often associated with effortless integration of incoming information and associative, heuristics-based thinking. In contrast, a negative mood typically implies extended inhibition of cognitive mechanisms engaged in information processing and ruminative thinking (see [20] for a review).

However, our results did not reveal an N400 modulation by mood that we predicted based on Pinheiro et al. [28]: an attenuated N400 response to meaningful sentences in a negative relative to positive mood. Instead, our results seem to concur with another result obtained by Pinheiro et al. [28]: an attenuation of the N400 response to within-category (i.e., unexpected word of the same semantic category) relative to between-category (i.e., unexpected word of a different semantic category) violations in the positive mood condition, together with a more pronounced N400 response to within-category violations relative to expected words in the negative mood condition. According to Pinheiro et al. [28], one interpretation is that moods may differently modulate the gradient of connections among different words in semantic memory, with a positive mood strengthening and a negative mood weakening them. Thus, participants in the present study might have perceived the critical words embedded in sentences as contextually unexpected yet not entirely meaningless, resembling the mechanisms engaged when processing within-category violations instead of the anticipated between-category violations.

Critically, consistent with our hypothesis, we observed differential effects of positive and negative moods on L1 processing only, with no mood effects for L2 processing. Additionally, the N400 response to meaningless sentences was reduced for L1 relative to L2 processing in a positive mood. Such results suggest that lexico-semantic processing in L2 may be more impervious to the effect of mood. This interpretation is consistent with recent evidence pointing to more effective implicit emotion regulation in L2 than L1. For instance, Morawetz et al. [18] observed that German–English bilinguals involuntarily displayed more effective emotion regulation mechanisms when presented with aversive pictures during L2 than L1 production. However, the L2 advantage disappeared when emotion regulation was explicitly invited through cognitive re-appraisal, suggesting that operating in L2 tends to activate regulatory mechanisms when emotional processing is spontaneous and implicit. Similarly, in the present study, participants read neutral L1 and L2 sentences and performed a semantic decision task, which did not explicitly require the activation of emotion regulation mechanisms. Therefore, we argue that increased emotion regulatory mechanisms triggered by L2 extend beyond L2 production [18], as our results suggest they are also active during L2 comprehension. This interpretation is in line with recent hemodynamic studies of bilingual speakers pointing to greater involvement of the amygdala (i.e., a subcortical structure ubiquitously involved in emotion processing and reinforcement) in L1 compared to L2 processing [84]. In sum, the N400 findings reported here suggest a decreased sensitivity to mood manipulation when unbalanced bilinguals process written content in L2 compared to L1.

### 4.4. Late Semantic Processing: LPC

LPC modulations have been linked to semantic integration and re-analysis, as well as the re-allocation of attentional, motivational, and evaluative resources [27]. Here, LPC mean amplitudes were greater for meaningful sentences in L2 than in L1 in the negative mood condition only. Consistent with our N400 effects (pointing to a dampened sensitivity to mood in L2) and previous evidence showing reduced emotional reactivity to L2 negative content [10,12,13], we propose that L2 processing triggers regulatory mechanisms protecting unbalanced bilinguals from adverse cognitive effects of a negative mood. Jończyk et al. [13], for instance, asked proficient immersed Polish–English bilinguals to assess the meaningfulness of L1 and L2 sentences ending in either affectively and semantically congruent or incongruent adjectives. They reported greater LPC amplitudes for L2 as compared to L1 negative sentences, a pattern very similar to that observed here in relation to mood manipulation. As originally proposed by Wu and Thierry [12], such an effect may relate to cognitive prevention, involuntarily activating a suppression mechanism upon encountering a potentially upsetting stimulus in L2, thereby inhibiting the full activation spread through the semantic network. This, in turn, would trigger re-evaluation mechanisms assessing the inhibited stimulus and engage memory-updating mechanisms. The same cognitive mechanisms could thus be triggered in our participants, with a negative mood context failing to modulate L2 sentence comprehension in the N400 range but nevertheless triggering re-evaluation processes to a greater extent in L2 than L1.

### 4.5. Response Accuracy

We observed equally accurate semantic judgements for meaningless and meaningful sentences in a positive mood, while meaningless relative to meaningful sentences were still identified more accurately in a negative mood. This supports the possibility that a positive mood led to more effective semantic judgements irrespective of the language of operation. Consistent with Wang et al. [81] and our electrophysiological data, such results suggest that a positive mood may prioritise the most important contextual information, at least when making rather cognitively untaxing semantic judgements.

### 4.6. Mood Manipulation

Consistently using an integrative approach to measure mood changes (see [85] for a review), we supplemented self-reported mood valence and arousal ratings (i.e., bipolar dimensions) with the results of PANAS [34], built on two unipolar dimensions of the positive affect (PA) and the negative affect (NA). Both mood valence and the PA/NA ratio consistently indicated that participants were responsive to mood manipulation, thus proving the affective evocativeness of the presented animated film clips. Interestingly, our correlational analyses also revealed that one’s susceptibility to positive mood manipulation may increase proportionally to their empathy level. A similar pattern was observed here for perceptual processing, as indexed by P1 responses.

## 5. Conclusions

Altogether, we found differential language-driven mood effects in four consecutive stages of bilingual word processing within a sentence context: lexical processing (as indexed by N1), lexico-semantic processing (as indexed by both N2 and N400), and semantic integration and re-analysis (as indexed by LPC). We argue that a negative mood may activate detail-oriented processing affecting lexical search in the language of operation requiring more attentional resources. We also propose that the between-language differences observed in the N400 and LPC ranges point to the activation of emotion regulation [18] and suppression [12] mechanisms during L2 processing, offering a form of cognitive protection against potentially disruptive effects of a negative mood. Our findings might have important implications for everyday situations, especially those conducive to negative moods (e.g., counselling or judiciary proceedings). Indeed, in such circumstances, operating in L2 might prove a useful emotion regulation strategy for bilinguals. This idea is consistent with clinical research showing that discussing traumatic experiences in L2 allows bilingual speakers to emotionally distance themselves from them [86].

## Figures and Tables

**Table 1:** Participants’ sociolinguistic data (means with 95% CI).

	Polish (L1)	English (L2)
Proficiency ^1^	n/a	91.33 (88.94, 93.72)
Proficiency ^2^	96.87 (94.91, 98.82)	90.13 (87.55, 92.71)
Dominance ^2^	57.63 (55.56, 59.71)	55.63 (52.60, 58.66)
Immersion ^2^	70.67 (65.81, 75.52)	69.10 (65.38, 72.82)
Age of acquisition ^2^	n/a	7.70 (6.50, 8.89)
Years of learning ^2^	n/a	17.53 (15.77, 19.30)
Frequency of expressing emotions ^2^	5.18 (4.70, 5.66)	4.14 (3.64, 4.63)

^1^ Based on the LexTALE test (the standardised LexTALE score) [32]. ^2^ Based on the language history questionnaire 3.0 (LHQ [33], as translated into Polish by Naranowicz and Witczak): the proficiency, dominance, and immersion scores (percentages), age of acquisition and years of use (years), and frequency of expressing emotions (1—never, 7—always).

**Table 2:** Participants’ characteristics (mean percentages with 95% CI).

Positive affect ^1^	63.70 (58.87, 68.54)	Agreeableness ^5^	81.80 (78.76, 84.84)
Negative affect ^1^	42.73 (39.73, 45.72)	Conscientiousness ^5^	72.87 (66.25, 79.48)
Handedness ^2^	81.10 (63.19, 99.01)	Neuroticism ^5^	57.60 (50.88, 64.31)
Empathy ^3^	46.47 (42.75, 50.19)	Openness to experience ^5^	77.00 (73.62, 80.38)
Depression ^4^	7.73 (5.61, 9.86)	Perspective-taking ^6^	68.45 (61.07, 75.83)
Anxiety ^4^	9.30 (6.27, 12.33)	Fantasy scale ^6^	70.95 (62.19, 79.72)
Stress ^4^	5.23 (2.81, 7.65)	Empathetic concern ^6^	77.50 (72.03, 82.97)
Extraversion ^5^	62.53 (55.84, 69.22)	Personal distress ^6^	49.76 (45.53, 53.99)

^1^ Based on the Positive and Negative Affect Schedule (PANAS [34], as translated into Polish by Fajkowska and Marszał-Wiśniewska [35]): positive affect (interested, excited, strong, enthusiastic, proud, alert, inspired, determined, attentive, and active) and negative affect (distressed, upset, guilty, scared, hostile, irritable, ashamed, nervous, jittery, and afraid). ^2^ Based on the handedness questionnaire [36] (as adapted from Oldfield [37]): left-handedness (−100–−28), ambidexterity (−29–48), and right-handedness (48–100). ^3^ Based on the Empathy Quotient [38] (as translated into Polish by Wainaina-Woźna): low (0–39%), average (40–64%), above average (65–78%), and high (79–100%) levels of empathy. ^4^ Based on the DASS-21 [39] (as translated into Polish by Makara-Studzińska et al.): normal (0–21%), mild (22–31%), moderate (32–48%), severe (49–64%), and extremely severe (65–100%) levels of depression, anxiety, and stress. ^5^ Based on the Big Five Inventory [40] (as translated into Polish by Strus et al. [41]): extraversion (talkativeness, activity, assertiveness vs. silence, passivity, reserve), agreeableness (kindness, trust, warmth vs. hostility, selfishness, distrust), conscientiousness (organisation, thoroughness, reliability vs. carelessness, negligence, unreliability), neuroticism (nervousness, moodiness, temperamentality vs. confidence, resilience), and openness to experience (imagination, curiosity, creativity vs. shallowness, imperceptiveness). ^6^ Based on the Interpersonal Reactivity Index [42] (as translated into Polish by Kaźmierczak et al. [43]): perspective-taking scale (“the tendency to spontaneously adopt the psychological point of view of others”), fantasy scale (one’s “tendencies to transpose themselves imaginatively into the feelings and actions of fictitious characters in books, movies, and plays”), empathetic concern scale (“other-oriented feelings of sympathy and concern for unfortunate others”), and personal distress scale (“self-oriented feelings of personal anxiety and unease in tense interpersonal settings”).

**Table 3:** The lexico-semantic properties of the critical words (means with 95% CI).

	Frequency ^1^	Word Valence ^2^	Arousal ^3^	Concreteness ^4^	Syllables ^5^	Letters ^6^
Polish (L1)	3.39 (3.32, 3.47)	4.36 (4.29, 4.42)	2.07 (2.00, 2.15)	6.59 (6.54, 6.65)	2.47 (2.36, 2.57)	6.93 (6.73, 7.14)
English (L2)	3.81 (3.72, 3.90)	4.43 (4.35, 4.50)	2.19 (2.11, 2.26)	6.43 (6.36, 6.51)	2.23 (2.14, 2.32)	7.27 (7.05, 7.48)

^1^ Based on SUBTLEX-UK [44] and SUBTLEX-PL [45] (the Zipf scale): 1—the lowest frequency, 7—the highest frequency. ^2^ Based on a norming study: 1—the word evokes strongly negative emotions, 7—the word evokes strongly positive emotions. ^3^ Based on a norming study: 1—the word makes me feel completely unaroused, 7—the word makes me feel highly aroused. ^4^ Based on a norming study: 1—the word is abstract, 7—the word is concrete. ^5^ Range = 2–4 syllables. ^6^ Range = 6–8 letters. Excluded words: Polish–English translation equivalents, polysemous words, cognates, and interlanguage homonyms and homographs (see [46]).

**Table 4:** Participants’ characteristics—all normative tests (means with 95% CI).

	Film Clips	Critical Words	Sentences	
Participants	50 Polish–English bilinguals (30/film clip)	121 Polish–English bilinguals (30/word)	325 Polish–English bilinguals (25/sentence)	210 English native speakers (30/sentence)
Gender ^1^	F: 50, M: 0, NB: 0	F: 101, M: 20, NB: 0	F: 259, M: 63, NB: 3	F: 121, M: 79, NB: 10
Age ^2^	21.19 (20.62, 21.76)	23.69 (23.36, 24.01)	20.69 (20.16, 21.22)	23.47 (20.85, 26.09)
L1 Proficiency ^3^	6.91 (6.80, 7.00)	6.84 (6.71, 6.98)	6.81 (6.60, 7.00)	6.88 (6.75, 7.00)
L2 Proficiency ^3^	5.44 (5.20, 5.68)	5.25 (4.96, 5.54)	5.43 (5.20, 5.69)	4.03 (3.49, 4.57)
Years of L2 learning ^2^	14.06 (12.83, 15.30)	15.75 (14.80, 16.70)	14.06 (13.02, 15.10)	8.81 (6.11, 11.51)

^1^ F—female, M—male, NB—non-binary. ^2^ The score in years. ^3^ Based on self-reported proficiency: 1—beginner, 7—native speaker.

**Table 5:** Results of the norming study on the experimental sentences (means with 95% CI).

	Meaningfulness ^1^		Probability of Encountering ^2^		Valence ^3^	
	Polish (L1)	English (L2)	Polish (L1)	English (L2)	Polish (L1)	English (L2)
Meaningful	6.43 (6.39, 6.47)	6.41 (6.38, 6.45)	5.25 (5.19, 5.32)	6.06 (6.01, 6.12)	4.09 (4.03, 4.16)	4.18 (4.11, 4.25)
Meaningless	1.50 (1.46, 1.54)	1.87 (1.83, 1.92)	1.49 (1.47, 1.52)	1.47 (1.44, 1.51)		

^1^ Based on a norming study: 1—totally meaningless, 7—totally meaningful. ^2^ Based on a norming study: 1—totally improbable, 7—totally probable. ^3^ Based on a norming study: 1—strongly negative, 7—strongly positive; to enable the assessment of the neutrality of the constructed sentence frames, 30 strongly positive and 30 strongly negative sentences adapted from Jończyk et al. [13] were used as filler sentences in each language.

**Table 6:** Mood Ratings from PANAS and the mood valence and arousal Scales (with 95% CI).

	Mood Valence	PANAS	Arousal
**Positive mood condition**			
Pre-experiment	5.14 (4.79, 5.48)	2.08 (1.83, 2.33)	3.50 (2.96, 4.04)
Post-experiment	5.95 (5.61, 6.30)	2.46 (2.21, 2.72)	3.86 (3.32, 4.40)
**Negative mood condition**			
Pre-experiment	5.64 (5.29, 5.98)	2.33 (2.08, 2.59)	3.73 (3.19, 4.27)
Post-experiment	3.05 (2.70, 3.39)	1.40 (1.14, 1.65)	4.36 (3.82, 4.90)

## Data Availability

The EEG data collected and analysed for the purpose of the current study are available from the corresponding author upon reasonable request.
